# Supplementary material for: By-Product Revalorization: Cava Lees Can Improve the Fermentation Process and Change the Volatile Profile of Bread
Source: Foods. 2022 May 7;11(9):1361. doi: 10.3390/foods11091361 (PMC9099486; doi:10.3390/foods11091361)
Supplement: Supplementary file 1 [file foods-11-01361-s001.zip › foods-1691812-supplementary.pdf]

foods

MDPI

Supplementary Material

Table S1. Main volatile compounds identified in Cava lees.

Compound	CAS-Num.	Odor [1]	Relative peak area abundance
ALCOHOLS			
3-methyl-1-butanol	123-51-3	fusel, oil, alcoholic, whiskey, fruity, banana	2.03 ± 0.62
1-Hexanol	111-27-3	sweet, resin, flower	0.40 ± 0.07
1-Octanol	111-87-5	moss, nut, mushroom, chemical	0.18 ± 0.02
2-Dodecanol	10203-28-8	-	0.16 ± 0.01
1-Decanol	112-30-1	fatty, waxy, floral, orange, sweet	0.34 ± 0.03
Phenethyl alcohol	60-12-8	floral, rose	1.07 ± 0.34
Total Alcohols			*4.18 ± 1.09*
ACIDS			
Acetic acid	64-19-7	pungent, sour	0.46 ± 0.11
Octanoic acid	124-07-2	oily, rancid	16.25 ± 4.74
Decanoic acid	334-48-5	sour, fatty	15.68 ± 5.84
Dodecanoic acid	143-07-7	mild, fatty, coconut, oily	1.63 ± 0.17
Hexadecanoic acid	57-10-3	-	0.35 ± 0.05
Total Acids			*34.37 ± 10.91*
ALDEHYDES			
Nonanal	124-19-6	piney, floral, citrusy, fat	0.53 ± 0.27
Decanal	112-31-2	beefy, musty, marine, cucumber	0.46 ± 0.07
Benzaldehyde	100-52-7	cherry, candy	0.38 ± 0.03
Total Aldehydes			*1.37 ± 0.37*
KETONES			
2-Undecanone	112-12-9	citrus, rose, iris	0.30 ± 0.01
Total Ketones			*0.30 ± 0.01*
ESTERS			
Isoamyl acetate	123-92-2	sweet, fruity, banana	1.13 ± 0.72
Hexyl acetate	142-92-7	sweet, fruity, herb	1.23 ± 0.39
Ethyl octanoate	106-32-1	fruity, floral	14.48 ± 0.35
Isoamyl octanoate	2035-99-6	sweet, oily, fruity, green, pineapple, coconut	2.74 ± 0.29
Octyl acetate	112-14-1	fruity, fatty	0.37 ± 0.16
Methyl decanoate	110-42-9	oily, wine, fruity, floral	0.16 ± 0.09
Ethyl decanoate	110-38-3	sweet, oily, nutlike	27.27 ± 5.58
Dodecyl acetate	112-66-3	sweet, fresh	0.90 ± 0.16
Decyl acetate	112-17-4	sweet, fatty, fruity	1.02 ± 0.61
Ethyl 9-decenoate	67233-91-4	fruity, fatty	0.86 ± 0.04
Phenethyl acetate	103-45-7	floral, rose, sweet, honey, fruity, tropical	1.54 ± 0.07
Ethyl laurate	106-33-2	sweet, waxy, creamy, floral	5.98 ± 0.61
Isoamyl decanoate	2306-91-4	waxy, banana, fruity, sweet, cognac, green	1.06 ± 0.05
Ethyl decanoate	628-97-7	waxy, fruity, creamy, vanilla, balsamic	0.58 ± 0.03

Ethyl 9-hexadecenoate	54546-22-4	-	0.90 ± 0.03
Total Esters			*60.22 ± 9.18*
TERPENES & NORISOPRENOIDS			
Vitispirane A	65416-59-3	floral, fruity, earthy, woody	2.63 ± 0.20
1,1,6-trimethyl-1,2-dihydronaphthalene (TDN)	30364-38-6	licorice	8.06 ± 0.08
β-Caryophyllene	87-44-5	sweet, woody, spice, clove	0.12 ± 0.01
Farnesol	4602-84-0	fresh, sweet, linden, floral, angelica	0.46 ± 0.02
Isocaryophyllene	118-65-0	woody, spicy	0.40 ± 0.02
Nerolidol	7212-44-4	floral, green, waxy, citrus, woody	0.27 ± 0.08
Total Terpenes			*11.94 ± 0.41*

[1] From [27]. The numbers indicate relative peak area abundance calculated against the total peak area eluted on the chromatogram, results are expressed as mean ± standard deviation of triplicates from total analysed samples.
